# Automated quantification of protein periodic nanostructures in fluorescence nanoscopy images: abundance and regularity of neuronal spectrin membrane-associated skeleton

**DOI:** 10.1038/s41598-017-16280-x

**Published:** 2017-11-22

**Authors:** Federico M. Barabas, Luciano A. Masullo, Martín D. Bordenave, Sebastián A. Giusti, Nicolás Unsain, Damián Refojo, Alfredo Cáceres, Fernando D. Stefani

**Affiliations:** 1 Centro de Investigaciones en Bionanociencias (CIBION), Consejo Nacional de Investigaciones Científicas y Técnicas (CONICET), Godoy Cruz 2390, C1425FQD Buenos Aires, Argentina; 2 Departamento de Física, Facultad de Ciencias Exactas y Naturales, Universidad de Buenos Aires, Pabellón 1 Ciudad Universitaria, C1428EHA Buenos Aires, Argentina; 3 Laboratorio de Neurobiología Molecular. Instituto de Investigación en Biomedicina de Buenos Aires (IBioBA), CONICET - Partner Institute of the Max Planck Society, Godoy Cruz, 2390, C1425FQD Buenos Aires, Argentina; 4 Laboratorio de Neurobiología, Instituto de Investigación Médica Mercedes y Martín Ferreyra (INIMEC, CONICET), Friuli 2434, 5016 Córdoba, Argentina; 5 Universidad Nacional de Córdoba (UNC), Av. Haya de la Torre s/n 5000, Córdoba, Argentina

## Abstract

Fluorescence nanoscopy imaging permits the observation of periodic supramolecular protein structures in their natural environment, as well as the unveiling of previously unknown protein periodic structures. Deciphering the biological functions of such protein nanostructures requires systematic and quantitative analysis of large number of images under different experimental conditions and specific stimuli. Here we present a method and an open source software for the automated quantification of protein periodic structures in super-resolved images. Its performance is demonstrated by analyzing the abundance and regularity of the spectrin membrane-associated periodic skeleton (MPS) in hippocampal neurons of 2 to 40 days *in vitro*, imaged by STED and STORM nanoscopy. The automated analysis reveals that both the abundance and the regularity of the MPS increase over time and reach maximum plateau values after 14 DIV. A detailed analysis of the distributions of correlation coefficients provides indication of dynamical assembly and disassembly of the MPS.

## Introduction

The advent of fluorescence nanoscopy techniques like Stimulated Emission Depletion microscopy (STED)^1,2^, Stochastic Optical Reconstruction Microscopy (STORM)^3^ and Photoactivatable Localization Microscopy (PALM)^4^, meant a revolution in the field of fluorescence-based imaging. Conceptually, they provide a spatial resolution only limited by the size of the fluorescent marker, while keeping the advantages of traditional fluorescence microscopy such as low invasiveness and high sensitivity and specificity. In practice, spatial resolution in biological samples has been limited by the photostability of fluorophores and the overall size of immune-labels to around 20 nm^5,6^. Remarkably, a recent breakthrough concept for the localization of single molecules using the minimum number of photons (MINFLUX) has the capacity of delivering routinely super-resolved images with 1 nm resolution^7^, which constitutes the ultimate physically meaningful limit for an optical technique. For these reasons, fluorescence nanoscopy is on its way to becoming the imaging standard for cellular biology.

Proteins frequently function in the form of regular or periodic, self-assembled supramolecular structures^8^, with typical sizes in the range of tens of nanometers. Super-resolution fluorescence imaging enables the visualization of such protein periodic structures in their natural environment, as demonstrated for example in the nuclear pore complex^5,9,10^. Furthermore, previously unknown periodic protein structures are being discovered by fluorescence nanoscopy imaging. A remarkable example is the unveiling of an actin/spectrin membrane-associated periodic skeleton (MPS) first observed in axons of hippocampal neurons in culture by Zhuang and colleagues^6^.

The MPS is composed of alternating ring-like structures of actin and spectrin, which underlie the circumference of axons and are repeated along the axonal shafts with a periodicity of around 190 nm. MPS are also present in dendrites and in cellular projections of mature oligodendrocites^11^. The high prevalence of this structure points towards functional or structural relevance in neurite development and physiology^11–13^. The MPS emerges during early stages of neuronal maturation. It appears after 2 days *in vitro* (DIV) in proximal regions of axons and then extends towards distal ends to become ubiquitous at approximately 7 DIV^13^. So far, quantitative analysis of the MPS has been performed by autocorrelation analysis with manual selection of regions-of-interest aiming to determine the “degree of spectrin periodicity” in different segments of axons^13^, for comparing the MPSs of axons and dendrites^12^ and for assessing the periodicity of bidimensional protein structures^11^. However, this approach has severe limitations. First, the regions of interest are handpicked with the risk of introducing subjective bias. Secondly, because such a task is highly time-consuming, only a relatively small number of neurons can be analyzed, limiting the statistical relevance of results. Quantitative studies of the MPS call for specific and automated image analysis tools that overcome these two major drawbacks, enabling the comparison between measurements obtained in different laboratories as well as the identification of subtle, but probably physiologically relevant effects.

Furthermore, the function of the MPS remains a matter of speculation and a significant amount of work is still necessary before a full picture is completed. It is yet unknown, for example, how the actin/spectrin-MPS of axons responds to physiological (i.e. electrical activity and neurotrophic factors) or pathological (i.e. excitotoxicity, hypoxia, inflammatory) stimuli. In general, advancing from observation and description, to deciphering mechanisms and function, requires systematic and quantitative studies involving larger numbers of images taken under varying experimental conditions and biological stimuli. This, even more, calls for reliable and automated image analysis tools for the automated quantification of periodic protein structures.

Here, we present a method and its implementation as an open-source image analysis tool for the automated quantification of the abundance and quality of protein periodic structures in images of biological samples. Its reliable performance is demonstrated by a quantitative examination of both STED and STORM super-resolved images of the MPS in spectrin-stained cultured hippocampal neurons at different developmental time points *in vitro*. The code, algorithms and main user functions are described and made available in a public repository.

## Results

### Working principle and steps of the analysis

**Figure 1:**
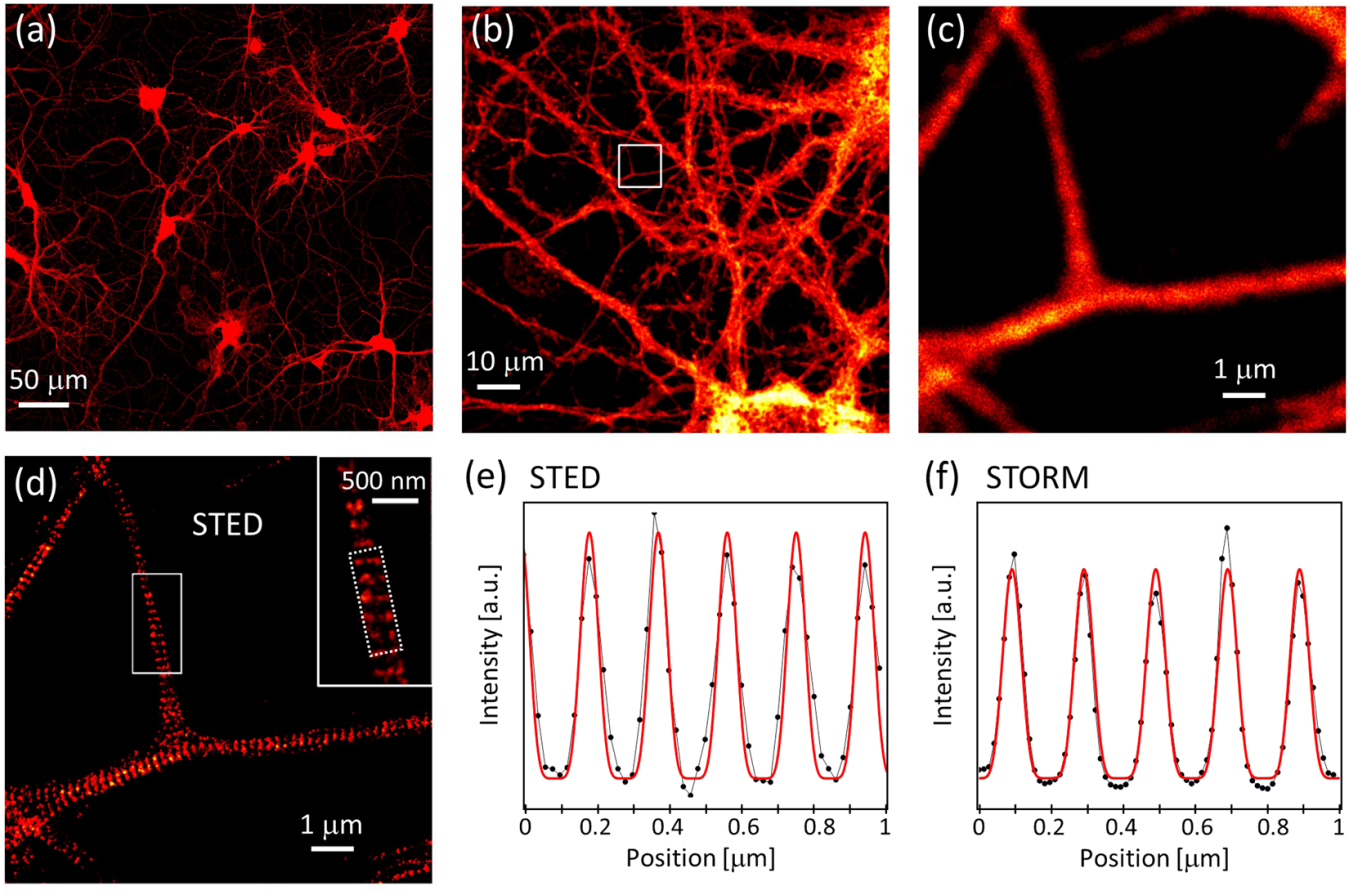
Hippocampal neurons cultured at 21 days *in vitro* fixed and immunostained against spectrin with ATTO 647 N. (**a**–**c**) Confocal images. (**d**) STED image of the area shown in (**c**). The sub-diffraction resolution of STED reveals the presence of MPS. (e, f) Representative profiles of the MPS obtained averaging 10 profiles of STED (**e**) and STORM (**f**) images. Both average profiles are satisfactorily fit using equation (1), with P = 6 and T_MPS_ = 190 nm.

Given a known periodic structure, the analysis consists of interrogating systematically the presence of the structure in images, by comparing subregions of the images to a reference pattern. As output, it provides the abundance and spatial distribution of the periodic structures, as well as a measure of the similarity to the reference pattern. We apply the methodology to the quantification of the spectrin MPS in cultured hippocampal neurons (Fig. 1a–c), which is clearly visible in both STED and STORM images of mature neurons (STED example in Fig. 1d). Below we describe each step of the analysis.

#### Definition of the reference pattern

The first step in the analysis is the definition of the reference periodic pattern to look for in the images. Since the final spatial resolution and image quality may vary for different nanoscopy methods, two reference patterns must be defined in our case, one for the analysis of STED images and another one for the analysis of STORM images. For this purpose, we computed the average of 10 representative profiles of the MPS as measured by STED and STORM (Fig. 1e,f) and searched for a suitable analytical function to represent it. The average MPS profiles of both STED and STORM images were satisfactorily fitted by the following function (Fig. 1e,f):

1 $$f(x)=A+B\,{\sin }^{{\rm{P}}}[\frac{\pi }{{{\rm{T}}}_{{\rm{M}}{\rm{P}}{\rm{S}}}}x+\phi ]$$

where $A$, $B$, ${{\rm{T}}}_{\mathrm{MPS}}$ and ${\rm{\phi }}$ represent the baseline, amplitude, period and phase of the MPS. The power ${\rm{P}}$ modulates the sharpness of the MPS features. We found best fits using ${\rm{P}}=6$ and ${{\rm{T}}}_{\mathrm{MPS}}=190$ nm.

#### Detection of biological material

**Figure 2:**
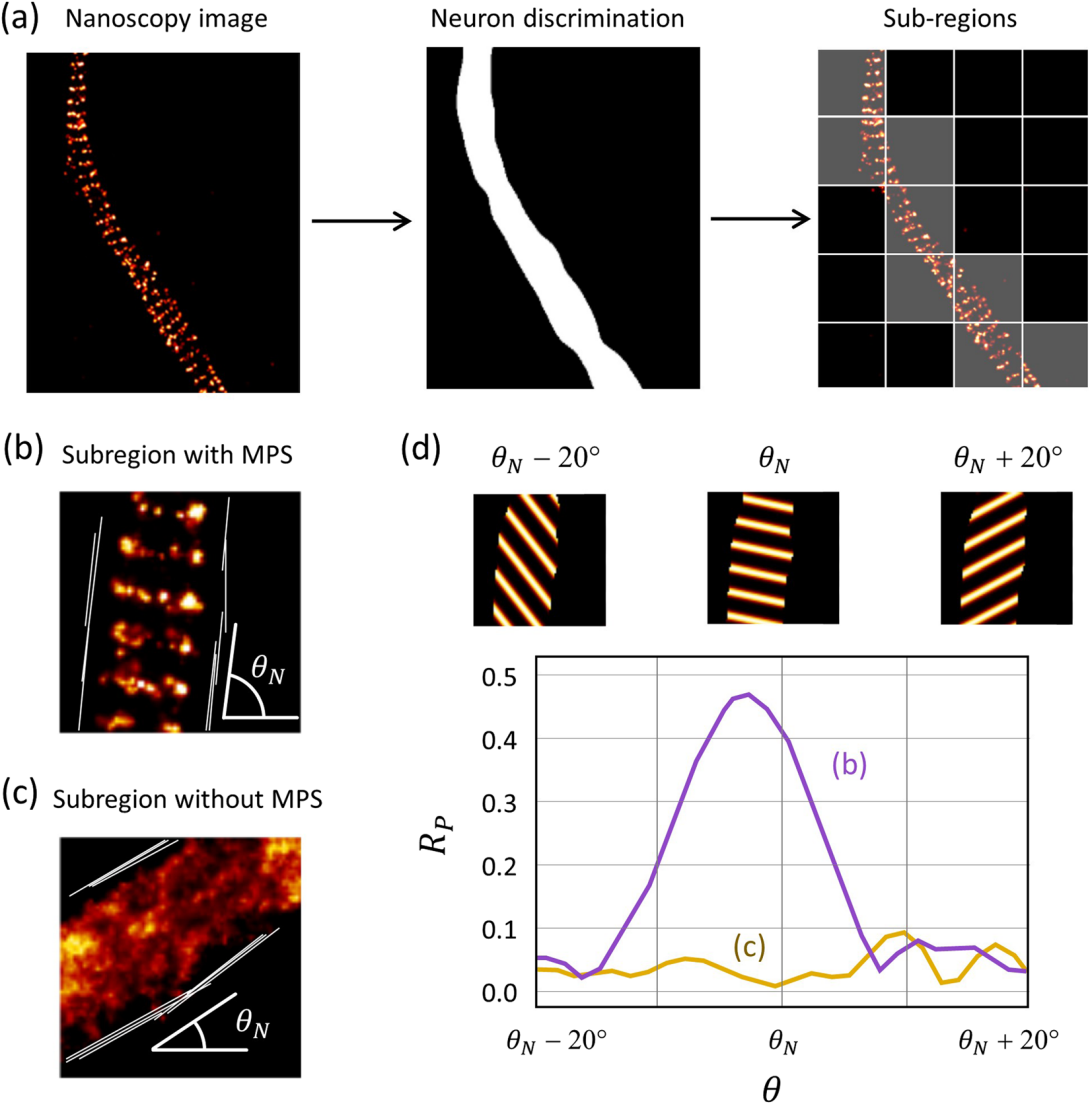
Workflow of the automated quantification of periodic structures exemplified with the MPS of a cultured hippocampal neuron imaged by STORM. (**a**) First, the regions containing neuronal material are identified using a suitable Gaussian filter and intensity threshold on the nanoscopy image (neuron discrimination). Then, the image is divided in subregions of predefined size (1 μm × 1 μm in this case) which are then catalogued as containing or not neuronal material. (**b,c**) The direction of the axon/dendrite ${\theta }_{N}$ is determined on each subregion. (**d**) The two-dimensional Pearson correlation coefficient ${R}_{P}$ is computed between each subregion and the reference pattern, within the area of neuronal material and for a range of directions $\theta$ and phases $\phi$ of the reference pattern. Here, the maximum value of ${R}_{P}(\phi )$ vs. $\theta$ is shown for the subregions shown in (**b**) and (**c**).

In order to analyze only meaningful regions of the nanoscopy images, an auxiliary image is generated where each pixel is classified as containing biological (neuronal) material or not. This is achieved by two steps, each one controlled by one user-defined parameter. First, a Gaussian filter of width ${\sigma }_{{GF}}$ is applied to the STED or STORM images in order to smooth out super-resolved features and small bright spots usually present in the cultures that should be considered as background. Then, the regions of the image containing labeled neuronal material are identified using an intensity discrimination threshold ${I}_{{Th}}$. All pixels with intensity above this threshold are considered as part of a neuron. A binary image indicating neuron presence at each pixel is built. We call it the *neuron discrimination image* (Fig. 2a).

Values of ${\sigma }_{{GF}}$ between 100 and 150 nm, and intensity thresholds ${I}_{{Th}}$ equivalent to 0.5–0.8 standard deviations above the average image intensity allowed to effectively identify the neuronal material on both STED and STORM images.

#### Image segmentation

Next, the image is segmented into subregions where the presence of the MPS will be interrogated locally. This is done in squared sub-regions whose size ${L}_{{sr}}$ can be defined by the user. In principle, a smaller subregion size is desirable to attain a higher spatial resolution of the analysis. But in order to allow a reliable identification of the MPS, the subregion size must be kept large enough so as to accommodate a few periods. We found a value of ${L}_{{sr}}$ = 1 μm suitable for the 190 nm period of the MPS.

#### Determination of axon/dendrite orientation

The algorithm must search for the presence of the MPS with *a priori* unknown direction $\theta$ and phase $\phi$, inside axons and dendrites, which are themselves directional structures. The automated search for the MPS is facilitated by estimating first the axon/dendrite direction. This is achieved by detecting linear intensity edges inside each subregion of the *neuron discrimination image* using a progressive probabilistic Hough transform algorithm^14^ (Fig. 2b,c). Then, a cluster analysis of the directions of the detected intensity edges is performed. If there is one main cluster with more than 50% of the occurrences, then the direction of the axon/dendrite ${\theta }_{N}$ is estimated as the mean value of that cluster. If no cluster fulfills this condition, that particular subregion is discarded from the subsequent analysis because a proper fit of the MPS is not possible when the axon/dendrite has a large curvature or more than one axon/dendrite is present. Another reason to choose a small subregion size in the case of the MPS is to keep the fraction of such subregions to a minimum, particularly when imaging high-density cultures where axons/dendrites frequently cross each other.

*Interrogating the presence of the target periodic structure*. The presence of the MPS is assessed in each subregion of the super-resolved image, by computing the pixel-by-pixel two-dimensional Pearson correlation coefficient (${R}_{P}$) with the reference pattern, only within the neuronal area:

2 $${R}_{P}=\,\frac{{\sum }_{m}{\sum }_{n}({I}_{mn}-I)({P}_{mn}-P)}{\sqrt{{\sum }_{m}{\sum }_{n}{({I}_{mn}-I)}^{2}{\sum }_{m}{\sum }_{n}{({P}_{mn}-P)}^{2}}}$$

where ${I}_{{mn}}$ and ${P}_{{mn}}$ are the intensities of the pixel with coordinates (*m*, *n*) of the subregion image and the reference pattern, respectively. $\langle I\rangle$ and $\langle P\rangle$ are the intensity averages of the image and the pattern, respectively. The coefficient is computed only over pixels corresponding to neuronal mass, determined from the neuron discrimination image. ${R}_{P}$ takes values between 0, for totally uncorrelated images, and 1 when both images are identical up to an intensity scaling factor. These extreme, ideal values are never reached in practice, but a clear maximum of ${R}_{P}$ is expected if the MPS is present and the reference pattern has the correct orientation $\theta$ and phase $\phi$. Since the orientation and phase of the MPS are *a priori* unknown, ${R}_{P}$ must be computed for each sub-region using all possible combinations of ($\theta$, $\phi$).

Taking advantage of the pre-analysis of axon/dendrite orientation, and the fact that the MPS is structured perpendicularly to the axon/dendrite axis, $\theta$ was scanned over a range of ±20° around the predetermined axon/dendrite direction ${\theta }_{N}$ (Fig. 2d). This was achieved by applying a rotation of $\theta$ to the reference pattern function (eq. 1). The phase $\phi$ was scanned over its full range of 2 $\pi$. The maximum value of ${R}_{P}(\phi )$ is plotted as a function of $\theta$ in Fig. 2d for the example subregions of Fig. 2b,c. The method is effective to detect the presence of MPS. Subregions where the MPS is present show a clear maximum ${R}_{P}$ near ${\theta }_{N}$, while subregions absent of MPS do not. (Fig. 2d). The maximum value of *R*_*P*_(*θ*, *φ*) obtained is stored as characteristic of the subregion.

### Test of performance and calibration for the automated detection

**Figure 3:**
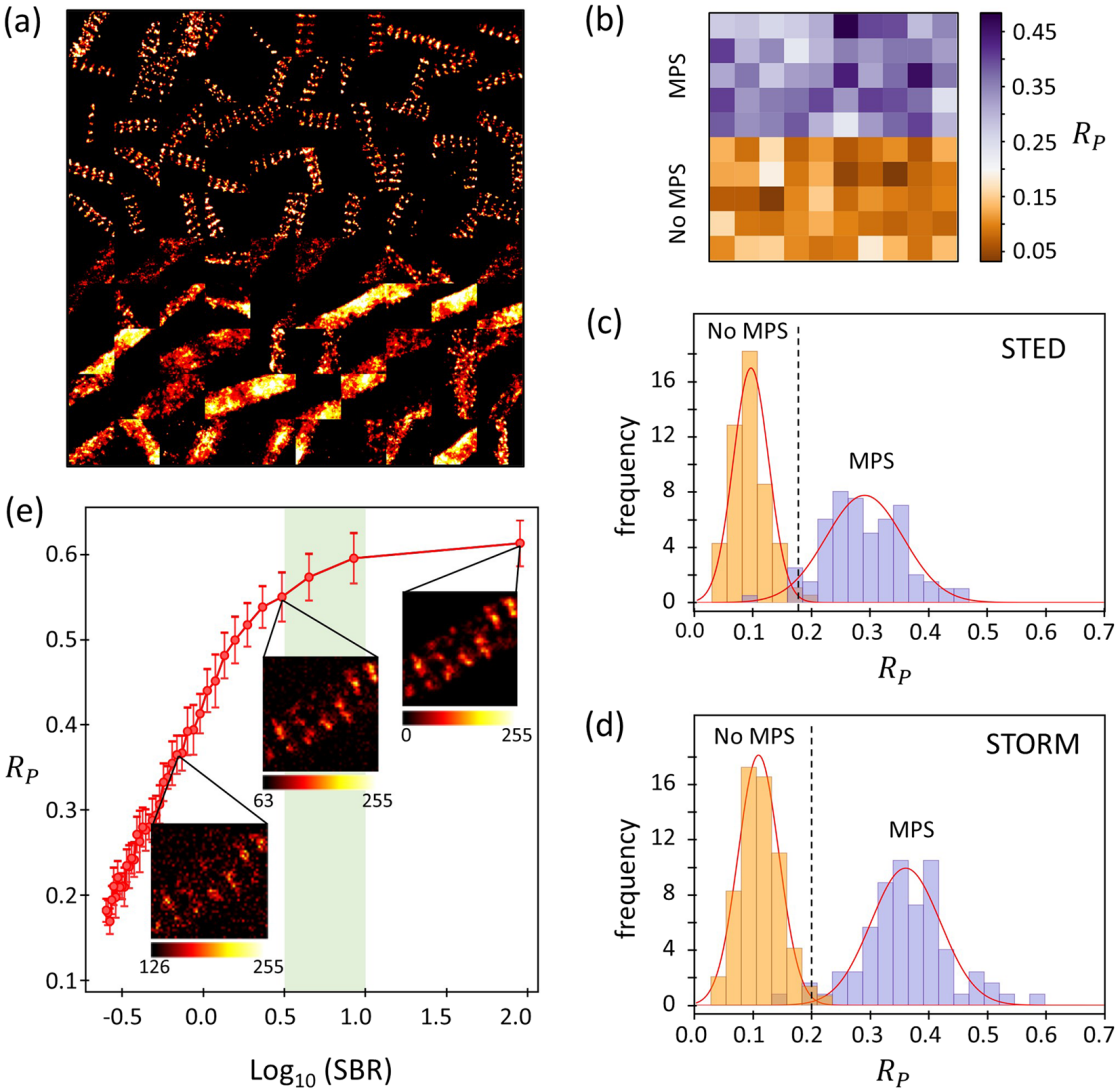
Performance of the automated detection algorithm to discern the presence of the MPS. (**a**) Image composed of 100 handpicked subregions (1 μm × 1μm each) of STORM images of axons and dendrites. The 50 subregions on the top half were selected for evidently showing the MPS, whereas the 50 on the bottom half were selected for the absence of MPS. (b) Image showing the maximum ${R}_{P}$ obtained for each subregion of (**a**) as the intensity values. (**c**,**d**) Distributions of ${R}_{P}$ obtained from subregions not showing MPS and clearly showing MPS, handpicked from STORM (**c**) and STED (**d**) images. The vertical dotted lines show the value $\overline{{R}_{P}}+2.5\,\sigma$ for the distributions of ${R}_{P}$ of subregions without MPS. (**e**) Dependency of ${R}_{P}$ on signal to background ratio (SBR) obtained from the analysis of simulated images. Error bars indicate ± one standard deviation of 10 simulations. The shaded green area shows the range of SBR typical of STED and STORM images.

Figure 3a shows an image composed of 100 STORM images, each one 1 × 1 μm^2^ and manually selected so that the 50 images at the top clearly exhibit the spectrin MPS, while the 50 images at the bottom do not. The result of the analysis of this test image is shown in Fig. 3b, where the maximum ${R}_{P}$ of each subregion is shown with a color scale. Clearly, the algorithm effectively recognizes the presence of the MPS; the two sets of images are discernable with a threshold value of ${R}_{P}$ between 0.18 and 0.24.

Setting an optimum threshold value of ${R}_{P}$ for the automated detection of the MPS requires an analysis of the distributions of maximum ${R}_{P}$ obtained from a large number of subregions with and without MPS, under the different imaging conditions. Figure 3c,d show the distributions of maximum ${R}_{P}$ obtained from the analysis of subregions handpicked with and without spectrin MPS, from STORM and STED images. The distributions are all nearly Gaussian, with slightly different averages ($\bar{{R}_{P}}$) and standard deviations ($\sigma )$ for STED and STORM. In this particular case, STORM delivers a slightly better discrimination of the two populations, probably due to its slightly superior resolution, a more favorable combination of primary and secondary immunolabeling and fluorophore performance. On the other hand, STED acquisitions are in general considerably faster than STORM (~s vs ~min), hence making STED imaging more convenient for acquiring large number of images in order to perform statistical analysis.

From the analysis of these distributions it is possible to set a threshold value of ${R}_{P}$ to detect the presence of the MPS with a predefined probability of false positives or false negatives. In this study we chose to limit the probability of false detections of the MPS to 1%. Therefore, we used a threshold at ${R}_{P}=\overline{{R}_{P}}+2.5\,\sigma$ of the distributions in the absence of MPS, which corresponds to ${R}_{P}=0.2$ for STORM and ${R}_{P}=0.17$ for STED images. These thresholds lead to 0.35% of false negatives for STORM, 4% of false negatives for STED imaging.

It is also interesting to probe the robustness of the method to detect the MPS. For this purpose we performed the analysis on simulated images where the immunolabeling density and unspecific labeling were adjusted to reproduce the experimental images, and the signal to background ratio (SBR) was varied in a controlled manner. Figure 3e shows the average value of ${R}_{P}$ obtained from the analysis of simulated images with different SBR. Each data point corresponds to the average of 10 simulations, and the error bars indicate one standard deviation. The SBR of our STED and STORM images ranged between 3 and 10. Such variations in SBR lead to an uncertainty of 10% in ${R}_{P}$.

### Automated batch analysis

**Figure 4:**
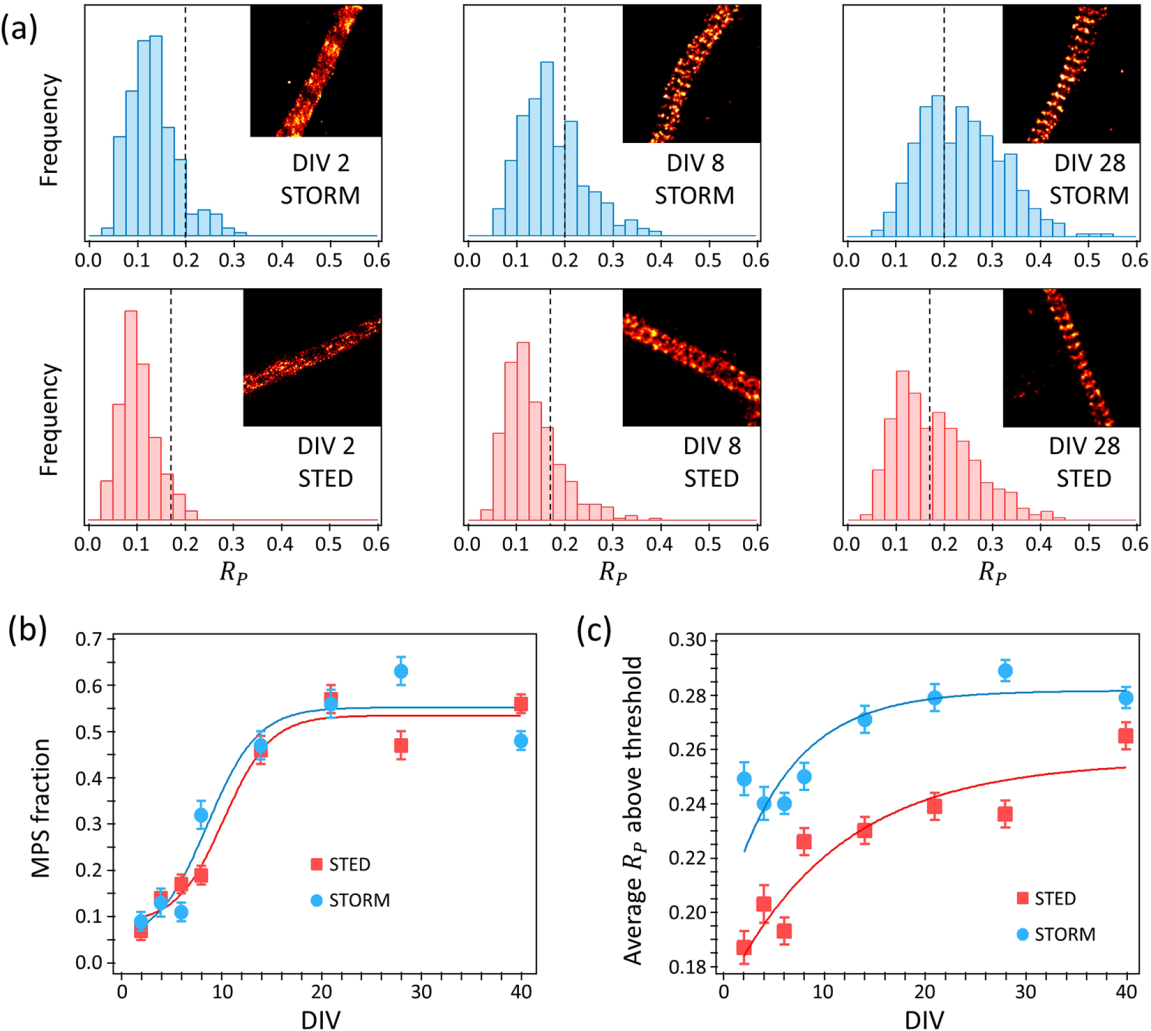
Evolution of the MPS vs. DIV. (**a**) Example STORM and STED images of the MPS neurons of 2, 8, and 28 DIVs, and the corresponding distributions of ${R}_{P}$. The vertical line indicates the threshold used for the discrimination of the MPS. (**b**) Fraction of subregions of STED and STORM images showing the MPS as a function of DIV. Lines are sigmoidal fits retrieving half-height times of 8.6 DIV (STORM) and 10.2 DIV (STED). (**c**) Average value of ${R}_{P}$ above threshold for STED and STORM images of neurons for DIVs from 2 to 40. Lines are exponential fits aiming only to reveal the increasing trend.

Once the threshold is chosen, batch analysis of a large number of images obtained under identical experimental conditions can be performed. Figure 4a shows example STORM and STED images of the spectrin MPS of hippocampal neurons of 2, 8 and 28 DIVs, alongside with their corresponding distributions of ${R}_{P}$. At DIV 2 the distributions of ${R}_{P}$ obtained from the STED and STORM images are practically identical to the distributions of ${R}_{P}$ values obtained from hand-picked images lacking the MPS. As the DIVs progress, the MPS becomes more evident and the fraction of subregions displaying values of ${R}_{P}$ above threshold increases. Figure 4b shows the quantification of the abundance of the spectrin MPS in STED and STORM images of hippocampal neurons from 2 to 40 DIV, computed as the fraction of subregions with ${R}_{P}$ above threshold. Both STED and STORM imaging reveal practically identical evolutions of the abundance of the spectrin MPS as a function of the DIV, reaching a maximum plateau after 14 DIV. Sigmoidal fits retrieve half-height times of 8.6 DIV for STORM and 10.2 DIV for STED.

Remarkably, the distributions of ${R}_{P}$ are not bimodal with mean values corresponding to the presence and absence of MPS. Not even for stages where the MPS has been fully established like DIV 28 or DIV 40 (e.g. Fig. 4a). This shows that the regularity of the MPS increases during neuronal development. The average value of ${R}_{P}$ of all subregions above the threshold serves as a measure of the similarity of the MPS to the reference, ideal pattern, i.e. a measure of the MPS regularity. Figure 4c shows the evolution of the average ${R}_{P}$ above threshold as a function of DIV. In both STORM and STED images the regularity of the MPS increases with DIV, reaching a plateau after 14 DIV. The plateau value of the STORM images was found to be 20% higher than for the STED images. Both plateau values are considerably lower than the corresponding average of handpicked images showing the MPS. The fact that even after complete neuronal maturation, the MPS is not present in all axonal or dendrite segments, nor is fully regular, is a strong indication of an underlying dynamical assembly and disassembly of the structure.

### Open source code and executable programs

The source code, Python executable programs with graphical user interfaces, and detailed documentation are maintained at a public repository in https://github.com/cibion-conicet/Gollum. The software set is entirely written in Python 3 with Qt as the graphical user interface (GUI) framework, making it cross-platform. It is composed of two Python executable programs which make use of the same underlying algorithms but serve different purposes: *Gollum* and *Gollum Developer*.

**Figure 5:**
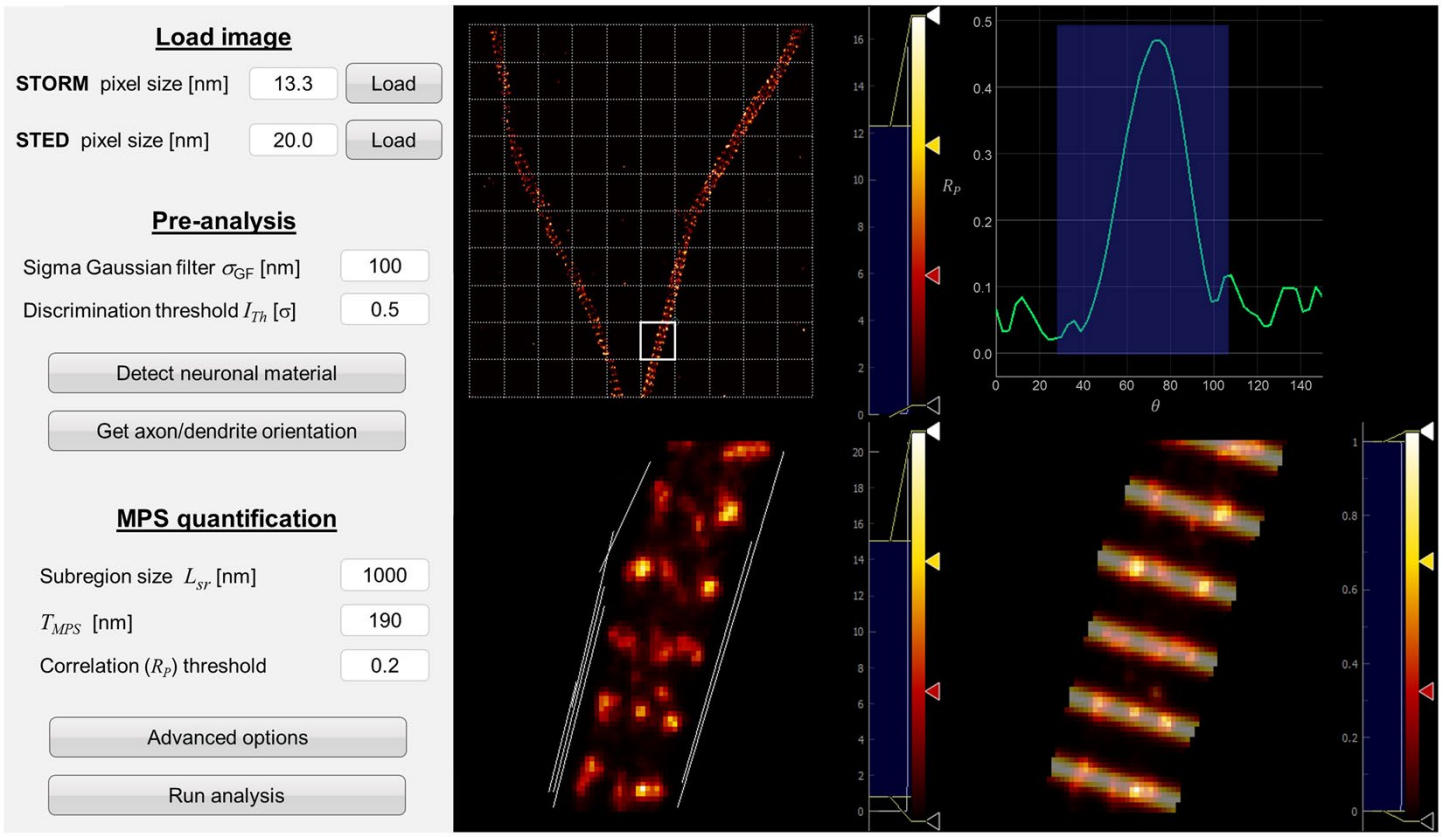
Gollum Developer graphical user interface (GUI). The parameters of the algorithm can be selected and each part of it can be run individually (left). A live display of the image and of the selected regions (right) allows to explore different parameters and to tune and identify optimal combinations for a given experiment.

Before performing a batch analysis of a large number of images, *Gollum Developer* (Fig. 5) is used for setting the analysis parameters so that the target periodic structure is identified reliably. These parameters are: i) pixel size of STED and STORM images, ii) the neuron discrimination settings ${\sigma }_{{GF}}$ and ${I}_{{Th}}$ iii) the sub-region size ${L}_{{sr}}$, iv) the period of the MPS, which in our case was maintained fixed at 190 mn, v) the sampling range and step for in *θ* and $\phi$. Some of these parameters can be modified directly from the GUI for faster testing (Fig. 5).

Naturally, the reference periodic pattern can be arbitrarily defined to search for different biological structures, with linear or cyclic periodicity. We have used an analytical expression for the reference pattern (eq. 1), but it is also possible to use reference patterns defined numerically. The parameters displayed in the GUI can be modified as well according to experimental needs or user preference.

The GUI also shows the loaded super-resolved image in the upper left view, overlaid with the sub-region divisions, in this case on a square grid of 1 × 1 µm^2^ unit cells. Individual subregions can be selected with mouse or keyboard, and analyzed. The selected subregion is highlighted in the original image and displayed in larger size on an individual graph (lower-left panel). The “Run correlation analysis” button triggers the analysis of the selected subregion. The detected linear intensity edges are shown. In the upper right, the maximum value of ${R}_{P}(\phi )$ is plotted as a function of *θ* over the full range. Knowing that the MPS structure evolves along the axon/dendrite orientation, *θ* can be scanned over a smaller range centered at the estimated axon/dendrite direction, defined by the used and shown shaded in blue. The maximum Pearson value within this range is taken as the characteristic value of the subregion. In the lower right view, the data is overlaid with the reference pattern for the combination of $\phi$ and *θ* that maximizes the Pearson value, so that the user can verify the effectivity of the identification. In this way, the user can test the reliability of analysis on hand-picked subregions and optimize the analysis parameters, including the definition of the reference pattern.

Once the analysis parameters are set, the user must switch to *Gollum* executable program, which performs automatic bulk processing of images. The detailed usage guidelines for *Gollum* are described in the documentation. The final output is the list of ${R}_{P}(\phi )$ values of all neuronal subregions, including the fraction of subregions where the periodical structure was positively identified. Another useful output is a binary image with value zero in all pixels or subregions absent of MPS, and value 1 in all pixels or subregions where MPS was detected. The analysis of 15 images, 1500 subregions, takes ~ 120 s running on an Intel i5–4440 CPU.

MS Windows executable versions of Gollum and Gollum Developer, with all necessary Python libraries embedded are available upon request.

## Discussion

We have presented an automated method, and its corresponding open source software, for the quantification of periodic protein structures in fluorescence nanoscopy images. It consists of analyzing subregions of the original image, testing the presence of a predefined target periodic pattern by computing the two-dimensional Pearson correlation. After calibration using handpicked subregions, batch analysis can be performed cataloguing each subregion for the presence or absence of the target periodic structure. Furthermore, the quality of the observed pattern can be addressed by quantitative analysis of the correlation coefficient.

As demonstration example, we have quantified the abundance and regularity of the spectrin membrane-associated periodic skeleton (MPS) of cultured hippocampal neurons during development, from 2 to 40 DIV, imaged by both coordinate stochastic (STORM) and coordinate targeted (STED) nanoscopy. The analysis of STED and STORM images retrieves consistent results, revealing a similar evolution of the MPS. The abundance and the regularity of the MPS increase over time and reach maximum plateau values after 14 DIV. A detailed analysis of the distributions of correlation coefficients indicate the presence of MPS of variable regularity. Also, even after full maturation, the MPS is present only in 50% of the axon/dendrite volume. Altogether these results are a strong indications of an underlying dynamical assembly and disassembly of the MPS.

Although it was not used in this study, the image segmentation of the method naturally offers the possibility of analyzing the presence and quality of periodic structures as a function of position. The spatial resolution of such analysis will depend on the particular characteristics of the periodic pattern; in this case it was 1 μm.

Finally, it is important to note that the method (and its open-source code) is highly versatile. It is applicable to practically any periodic structure simply using a suitable reference pattern, including cyclic structures such as the nuclear pore complex. Supramolecular protein structures typically have well defined geometries; the MPS has been found to have a period of 190 nm ubiquitously in practically all types of neurons^11,12^. For this reason we have chosen to keep fixed any fitting parameter related to the periodic structure, and scanned only orientational and phase parameters to interrogate the presence and quality of the structures. Nevertheless, it is straightforward to modify the algorithm to scan additional parameters, including characteristics of the protein periodic structure.

As fluorescence nanoscopy becomes the imaging standard in cellular biology, quantitative and automated analysis tools will become necessary to perform statistical studies of images obtained under different conditions, aiming to decipher the function biological nanostructures. Such tools are necessary not only for the efficient analysis of large number of images, but also to enable the quantitative comparison and complementation of data obtained in different laboratories. We believe that our method and future derivations of it will be frequently used for functional studies of periodic protein structures visualized in their natural environment through fluorescence nanoscopy.

## Methods

### Primary hippocampal neuronal cultures

CD1 mice were provided by our Specific Pathogen Free Animal Facility. All procedures were approved by National Department of Animal Care and Health (SENASA, Argentina) and were in compliance with the general guidelines of the National Institute of Health (NIH, USA). Primary hippocampal neurons were prepared as previously described^15,16^. Briefly, hippocampi from CD1 mouse embryos (E16.5–17.5) were dissected and a neuronal suspension was prepared through Trypsin digestion and mechanical disruption of the tissue. Neurons were plated at a density of 125 cells/mm^2^ and maintained in Neurobasal-A medium with 2% B27 and 0.5 mMGlutaMAX-I (Gibco) at 37 °C and 5% CO_2_.

### Immunofluorescence

Neurons were simultaneously fixed and permeabilized in PHEM buffer (60 mM PIPES, 25 mM HEPES, 5 mM EGTA, 1 mM MgCl_2_) containing 0.25% glutaraldehyde, 3.7% paraformaldehyde, 3.7% sucrose, and 0.1% Triton X-100, for 20 min at RT. Samples were quenched with 0.1 M glycine in PBS for 15 min and blocked for 1 h in 5% BSA solution in PBS containing 0.01% Triton X-100. Purified Mouse Anti- β-Spectrin II primary antibody (Clone 42/B-Spectrin II, BD Biosciences) was diluted 1:400 in blocking solution and incubated with the samples overnight at 4 °C. For STED microscopy, an anti-mouse secondary antibody (1:250) conjugated to Atto647N (Sigma) was used andcoverslips were mounted in slides with a home-made mounting media based in Mowiol (2.4% Mowiol 4–88 (poly(vinyl alcohol), Sigma) and the antifade reagent DABCO (2.5% w/v, 1,4-diazobicyclo[2.2.2]octane, Sigma), as described previously^17^, which provided a suitable refractive index. For STORM microscopy, the secondary antibody (1:750) was conjugated to Alexa647 (Life Technologies).

### STORM imaging

The STORM microscope was custom-built around an Olympus IX-73 inverted microscope operating in wide-field epifluorescence mode. A 642 nm 1.5 W laser (MPB Communications 2RU-VFL-P-1500–642) was used for fluorescence excitation and a 405 nm 50 mW laser (RGB Photonics Lambda Mini) for fluorescence re-activation. The lasers were combined with a dichroic mirror (CM01–427, Semrock), magnified and then focused to the back focal plane of the oil immersion objective Olympus PlanApo 60x NA 1.42. A dichroic mirror (Di03-R 405/488/532/635-t1 25 × 36, Semrock) and a band-pass filter (ET700/75 m, Chroma) were used for decoupling of the fluorescence emission of the sample from the laser excitation. Further blocking of the illumination lasers was performed with a multi-edge notch filter (NF03–405/488/532/635E-25, Semrock). The emission light was expanded with a 2x telescope so that the pixel size of the EMCCD camera (Andor iXon3 897) would match the optimal value for single-molecule localization. The camera and lasers were controlled with a custom software developed in the laboratory and described in^18^.

Cells cultured on 18 mm coverslips were placed in a holder and imaging was perform in a 50 mM Tris pH = 8, 10 mM NaCl buffer. The imaging buffer was supplemented with 10% w/v glucose, 100 mM beta-mercaptoethanol, 1 μg/mL glucose oxidase (Sigma-Aldrich) and 0.5 ug/mL catalase (Sigma-Aldrich) as oxygen scavenging system.

Prior to STORM imaging, conventional fluorescence images of the region of interest were acquired by setting the excitation laser intensity to 1–5 W cm^−2^. STORM data acquisition was then started by changing the excitation laser intensity to 20–30 kW cm^−2^, thus inducing on-off switching of the fluorescent marker in the tens of ms time range, as required by the STORM technique. Throughout the whole acquisition, the activation 405 nm laser power was increased in steps whenever the density of single-molecule events decreased below ~1 molecule per μm^2^. Typically, it took 25000 frames at 20 ms of exposition time for each STORM acquisition. Data analysis and the rendering of the final super-resolved image were performed with ThunderSTORM software^19^.

### STED imaging

The STED nanoscope was home-built. For excitation of fluorescence, a linearly polarized pulsed (200 ps) laser at 640 nm (PicoQuant LDH-P-C-640B) operating at 20 MHz repetition was used. Light was coupled into a polarization maintaining single-mode fiber (Thorlabs P3-488PM-FC-5) using a fiber collimator (Schäffer + Kirchhoff 60FC-4-A7.5-01). Light exiting the fiber was collimated (f = 30 mm) in order to obtain a TEM00 excitation beam and circular polarization was adjusted using a broadband (400 nm–800 nm) quarter-wave plate (Thorlabs AQWP05M-600) and a broadband (400 nm–800 nm) half-wave plate (Thorlabs AHWP05M-600).

For STED, a linearly polarized pulsed (1 ns) laser at 775 nm was used (Onefive Katana HP). Light was coupled into a polarization maintaining single-mode fiber (Thorlabs P3-630PM-FC-5) using a fiber collimator (Schäffer + Kirchhoff 60FC-4-A11-02). STED light coming out of the fiber was then collimated (f = 30 mm) and sent through a 2π vortex phase plate (RPC Photonics VPP-1a). Circular polarization was adjusted using a broadband (690 nm–1200 nm) quarter-wave plate (Thorlabs AQWP05M-980) and a broadband (690 nm–1200 nm) half-wave plate (Thorlabs AHWP05M-980).

Excitation and STED beams were combined using a notch filter at 22 degrees (Semrock NF03-658-25) and a 5 mm short pass dichroic mirror (Chroma Z780sprdc) respectively. Lateral beam scanning was performed by a system composed of a home-made galvanometric scanner, a scanning lens (Leica) and a tube lens (Leica). For additional positional control and fine focusing, the sample was mounted on a XYZ piezoelectronic nanopositioning stage (Thorlabs NanoMax MAX311D/M). Light was focused to the diffraction limit with an objective of 1.4 NA (Leica HCX PL APO 100x/1.40-0.70 Oil CS).

Co-alignment of the excitation and STED foci was performed measuring scattering of 40 nm gold nanoparticles. A magnetically mounted pellicle beamsplitter coated for 400 nm–700 nm, 45:55 (R:T) (Thorlabs BP145B1) directed scattering light to a broadband (280 nm–850 nm) photomultiplier tube (Thorlabs PMM02).

STED wavelength was rejected from the detection path using a notch filter (Semrock NF03-785-25), fluorescence was selected using a bandpass filter (Semrock FF01-676/37-25). Light was then coupled (f = 50 mm) into a 25 μm, 0.1 NA multi-mode fiber (Thorlabs M67L01) connected to the photodetector (MPD PD-050-CTC-FC). Time gating of fluorescence photons was performed using a custom-made electronic board (MPI for biophysical Chemistry). The scanning and fluorescence count acquisition were computer controlled via an AD/DA board (National Instruments PCIe-6353) using the software ImSpector^20^.

### Numerical simulation

Simulated segments of a neurite presenting the MPS is created in four steps. First, a 2D matrix that is a rotation of the periodic function described in equation (1) is created, this is considered the ground truth and has a digital resolution of 1 nm. Second, the immunolabeling process is simulated by assigning a probability to each element of the matrix that is proportional to the intensity of the periodic function. A smaller probability of labeling is added throughout the neurite to simulate non-specific labeling. The final number of simulated fluorophores is adjusted in order to match experimental conditions. Third, the image of the simulated fluorophores is convoluted with a Gaussian function with FWHM = 40 nm simulating a typical super-resolution effective PSF. Finally, a Gaussian background with a given average and a 10% standard deviation is added, and Poissonian shot-noise is added to the resulting image accounting for the emission and detection process. The average of the Gaussian background contribution is varied to obtain images with different signal-to-background ratios (SBRs).
